# Perspective: Developing Future Veterans Affairs Scientists Through a Diversity, Equity, and Inclusion Program

**DOI:** 10.1089/heq.2023.0003

**Published:** 2023-05-26

**Authors:** Darius B. Dawson, Valerie Lewis, Terri L. Fletcher, Robert Hal Scofield

**Affiliations:** ^1^ Michael E. DeBakey VA Medical Center, Houston, Texas, USA.; ^2^ Baylor College of Medicine, Houston, Texas, USA.; ^3^ Oklahoma City VA Medical Center, Oklahoma City, Oklahoma, USA.; ^4^ University of Oklahoma Health Sciences Center, Oklahoma City, Oklahoma, USA.; ^5^ Oklahoma Medical Research Foundation, Oklahoma City, Oklahoma, USA.; ^6^ Langston University, Langston, Oklahoma, USA.

**Keywords:** research supplements, career development, mentoring

## Abstract

For >95 years, the Department of Veterans Affairs Office of Research and Development (ORD) has been improving the lives of Veterans and all Americans through health care discovery and innovation. Scientists and trainees from diverse backgrounds and life experiences bring different perspectives and creativity to address complex health-related problems, which helps to foster scientific innovation, improve quality of research, and advance the likelihood that underserved populations participate in and benefit from clinical and health services research. In this study, we will discuss our experiences in developing future scientists through mentored research supplements supported by ORD.

The Veterans Affairs (VA) Office of Research and Development's (ORD) ability to ensure innovations in health care for the benefit of our nation's Veterans is dependent on fostering a pool of highly talented motivated scientists from diverse backgrounds who will help advance VA's mission. To promote this goal, ORD created a funding opportunity in 2021 called the ORD Research Supplement to Promote Diversity. Through this program, funded VA investigators may apply for up to 2 years of funding to support an early-career scientist from an under-represented background. These supplements are a central step in ORD's career development pipeline and are meant to support early-career candidates as they prepare to apply for a VA Career Development Award.

To date, 32 supplements have been awarded to early-career scientists located at 24 VA Medical Centers nationwide. Research topic areas for funded supplements include pain/substance use disorders, health disparities research, remote delivery of care, diabetes, sleep disorders, suicide prevention, cardiovascular disorders, deployment health, and oncology, among others. ORD is committed to building strong relationships between senior and early-career scientists to encourage and foster the participation of minority scientists in VA research. The following perspectives highlight two mentee/mentor pairs who discuss their mentoring relationship, and what it represents to them personally and professionally.

Supplement awardees are listed in Table 1. More information on these cutting-edge research projects can be found at Diversity, Equity, and Inclusion Initiative (va.gov).

**Table 1.** Veterans Affairs Office of Research and Development Research Supplement to Promote Diversity Awardees

Mentee	Mentor	Location
FY 2021 Awardees		
Olamide Alabi, MD	Luke Brewster, MD	Atlanta VA Medical Center
Nadir Balba, PhD	Miranda Lim, MD, PhD	VA Portland Health Care System
Melissa Chinchilla, PhD	Alexander Young, MD	VA Greater Los Angeles Healthcare System
Darius Dawson, PhD	Terri L. Fletcher, PhD	Houston VA Medical Center
Marva Foster, PhD	D. Keith McInnes, ScD, MSc	VA Bedford Health Care System
Letitia Graves, PhD	Kath Bogie, DPhil	Cleveland VA Medical Center
Valerie Harris-Lewis, PhD	R. Hal Scofield, MD	Oklahoma City VA Medical Center
Kevin Ahmaad Jenkins, PhD	Amol Navathe, MD, PhD	Philadelphia VA Medical Center
Briana Mason, PhD	Gregory Collins, PhD	VA South Texas Health Care System
A'mie Preston, PSYD	Prasad R. Padala, MD, MS	Central Arkansas VA Medical Center
Shamira Rothmiller	Katie Hadlandsmyth, PhD	Iowa City VA Medical Center
FY 2022 Awardees		
Rehana Akter, PhD	Steven Kahn, MB, ChB	VA Puget Sound Health Care System
Shayna Brathwaite, MD	Nader Massarweh, MD, MPH	Atlanta VA Medical Center
Aryan Esmaeli, PhD	Clara Dismuke, PhD	VA Palo Alto Health Care System
Jason Flake, PhD	James LePage, PhD	VA North Texas Health Care System
Eric Knott, MD	Diane C. Lim, MD	Miami VA Health Care System
Robert Lane, PhD	Marianne Goodman, MD	Bronx, NY VA Medical Center
Brandy Martinez, PhD	Rajendra A. Morey, MD	Durham VA Medical Center
Vanessa Caridad Somohano, PhD	Lauren Denneson, PhD	VA Portland Health Care System
Michelle Tamplin, PhD	Isabella Grumbach, MD, PhD	Iowa City VA Medical Center
Timothy Troppoli, PhD	Ritchie Brown, PhD	VA Boston Health Care System
FY 2023 Awardees		
Delisa Brown, PhD	Lisham Ashrafioun, PhD	VA Finger Lakes Health Care System
Kevin Burt, PhD	Robert Mauck, PhD	Philadelphia VA Medical Center
Miguel de la Flor, PhD	Jason O'Connor PhD	South Texas VA Health Care System
Andrew Flores, PhD	Thomas Hnasko	San Diego VA Health Care System
Lily Lau, PhD	Anthony Chen, MD	VA Northern California Health Care System
Roberto Mendez, PhD	Alan Morrison, MD, PhD	Providence VA Medical Center
Diana Naranjo, PhD	Susan Zickmund, PhD	Salt Lake City VA Health Care System
Luis Rodriguez, PhD	Michael Beers, MD	Philadelphia VA Medical Center
Linda Diem Tran, PhD	Todd Wagner, PhD	VA Palo Alto Health Care System
Geneva Wilson, PhD	Charlesnika Evans, PhD, MPH	Edward Hines, Jr VA Medical Center
Julie Wu, MD, PhD	Shipra Arya, MD	VA Palo Alto Health Care System

VA, Veterans Affairs.

## Perspective 1: Darius B. Dawson, PhD and Terri L. Fletcher, PhD

As VA researchers, fostering health equity in mental and behavioral health treatment and advancing the lives of Veterans from racially minoritized groups is of utmost importance.^1^ Although working toward this goal can be personally challenging, it is alleviated by effective collaboration among researchers.^2,3^ Individually, our study has had different focuses: increasing access to tobacco cessation treatment for racially minoritized Veterans (Dawson) and increasing access to evidence-based treatments for Veterans with anxiety (Fletcher).

However, we embraced the opportunity to combine our research interests with the VA ORD Supplement to Promote Diversity to maximize the potential impacts of our research. Our supplement project uses a mixed-methods approach to examine the influence of cultural factors such as stigma and discrimination on anxiety symptom presentation, on accessing mental health treatment, and on participating in research. We anticipate impactful findings from the ORD supplement research that will inform the development of educational resources for researchers and providers detailing strategies to address stigma and discrimination that limit access to mental health care and prevent research participation among minoritized individuals.

Another important goal of the ORD supplement mechanism is to support researchers from racially minoritized groups. In addition to dealing with typical career demands, minoritized researchers must also deal with the effects of structural racism.^4^ We and others believe that achieving health equity requires diversifying the research field to attain unique perspectives on addressing societal and systemic issues impacting the health care of minoritized individuals.^5,6^ Each ORD diversity, equity, and inclusion (DEI) supplement candidate received salary support to conduct the research program and protected time to develop a Career Development Award application, which are critical to establishing a research career. We were also connected to other mentors and mentees within VA, creating an inclusive and diverse community of researchers working toward a shared mission.

The supplement also provided an opportunity to strengthen our professional partnership as a Black male mentee (Dawson) and a White female mentor (Fletcher). To develop a deeper understanding of how our backgrounds have shaped our approach to our research and everyday lives, we prioritized participation in DEI activities, guided by a research framework that details relational concerns in cross-race and cross-gender mentoring.^7^ We scheduled time to advance our understanding of DEI issues and discussed our past experiences with mentoring to create deeper understanding of our interactions, building a higher level of trust. Our study together has been incredibly fruitful due to our efforts in creating a strong mentoring relationship.

We plan to use the supplement project as pilot study for future grant applications to further our shared goal of improving health equity for Veterans with mental and behavioral health conditions and simultaneously enhancing both of our careers as researchers. Our experience with the ORD DEI supplement program serves as an example of the many benefits of supporting health equity research and minoritized early-career researchers.

## Perspective 2: Valerie Lewis, PhD and R. Hal Scofield, MD

R. Hal Scofield: I was raised in rural Texas at the end of the Jim Crow era in the South, or as historians might say, I am a “primary source.” Not only did I personally observe the inequities of life in small town Texas in the 1960s, they were definitively pointed out to me by my mother and father. He was the first lawyer in the county who accepted a Black American as a client in a civil matter. My mother asked the wife of a Black dentist from Dallas, Texas, to speak at the local women's club about not being able to find a restaurant that would serve her and her husband, when driving to Houston.

I could go on and on with the stories about inequities in my hometown but suffice it to say that I grew up with the notion that the way Black Americans were treated had to change. One of the best things about my job as a physician-scientist is bringing the next generation of scientists into medicine and research. Having an opportunity to mentor students from minority groups has enriched my own experience and gives me hope that we can continue to increase representation and diversity in medical research.

Valerie Lewis: Dr. Hal Scofield likes to share the story about how I was first hired as a research technician in his laboratory, before becoming his graduate student. He says I was the first person they interviewed for the position, and immediately after I left, he told my future laboratory mates, “Guess there's no reason to interview anyone else.” This story always makes me smile, because it is a pleasant reminder that he has believed in me since day one. Our relationship has only grown stronger since that time.

As a Black woman at a predominately White institution, I learned to develop a thick skin to deal with the microaggressions and subtle racism that I encountered. Hal, an older White man from rural Texas, has always given me a safe space to complain and simply “just feel.” As I have entered my postdoctoral career, I have become more driven to help increase diversity in biomedical research and in my research project. My mentor has been fully supportive as I have shifted my early career research project to focus on the functional genetics of Black lupus. He and I both understand that increasing representation in science and medicine is beneficial to both the minority scientist and physician, and more importantly, minority research subjects and patients.

Hal agrees with this sentiment wholeheartedly and showed his support by writing a pilot grant to fund scholars at our local Historically Black College and University, Langston University, to perform research at the Oklahoma City VA Medical Center. This grant was funded through VA, and I am the Program Manager. Through this program, we are sponsoring 11 students who will return each summer to work with VA faculty on various research projects. Our hope is to make a difference in these student's lives on the individual level, and to make a difference at a systematic level by increasing minority representation in medical research. At first glance, Hal and I may seem like an unlikely duo, but we make a great team.

## Abbreviations Used

DEI: diversity, equity, and inclusion
ORD: Office of Research and Development
VA: Veterans Affairs
